# Supplementary material for: Causal Relationship between Aging and Anorexia Nervosa: A White-Matter-Microstructure-Mediated Mendelian Randomization Analysis
Source: Biomedicines. 2024 Aug 16;12(8):1874. doi: 10.3390/biomedicines12081874 (PMC11351342; doi:10.3390/biomedicines12081874)
Supplement: Supplementary file 1 [file biomedicines-12-01874-s001.zip › Supplementary Figure/Figure S4/Singe SNP plot of MR analysis for TL to ubm-b-1548.pdf]

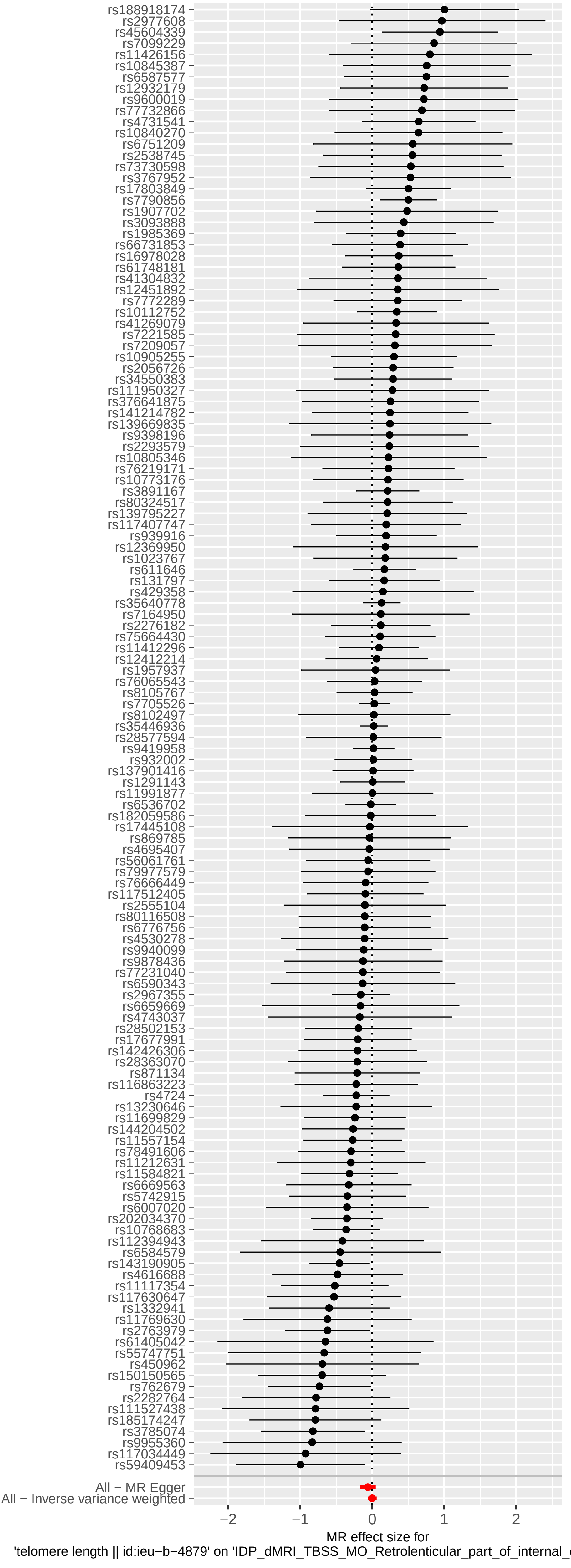

rs188918174
rs2977608
rs45604339
rs7099229
rs11426156
rs10845387
rs6587577
rs12932179
rs9600019
rs77732866
rs4731541
rs10840270
rs6751209
rs2538745
rs73730598
rs3767952
rs17803849
rs7790856
rs1907702
rs3093888
rs1985369
rs66731853
rs16978028
rs61748181
rs41304832
rs12451892
rs7772289
rs10112752
rs41269079
rs7221585
rs7209057
rs10905255
rs2056726
rs34550383
rs111950327
rs376641875
rs141214782
rs139669835
rs9398196
rs2293579
rs10805346
rs76219171
rs10773176
rs3891167
rs80324517
rs139795227
rs117407747
rs939916
rs12369950
rs1023767
rs611646
rs131797
rs429358
rs35640778
rs7164950
rs2276182
rs75664430
rs11412296
rs12412214
rs1957937
rs76065543
rs8105767
rs7705526
rs8102497
rs35446936
rs28577594
rs9419958
rs932002
rs137901416
rs1291143
rs11991877
rs6536702
rs182059586
rs17445108
rs869785
rs4695407
rs56061761
rs79977579
rs76666449
rs117512405
rs2555104
rs80116508
rs6776756
rs4530278
rs9940099
rs9878436
rs77231040
rs6590343
rs2967355
rs6659669
rs4743037
rs28502153
rs17677991
rs142426306
rs28363070
rs871134
rs116863223
rs4724
rs13230646
rs11699829
rs144204502
rs11557154
rs78491606
rs11212631
rs11584821
rs6669563
rs5742915
rs6007020
rs202034370
rs10768683
rs112394943
rs6584579
rs143190905
rs4616688
rs11117354
rs117630647
rs1332941
rs11769630
rs2763979
rs61405042
rs55747751
rs450962
rs150150565
rs762679
rs2282764
rs111527438
rs185174247
rs3785074
rs9955360
rs117034449
rs59409453
All − MR Egger
All − Inverse variance weighted
−2
−1
0
1
2
MR effect size for
'telomere length || id:ieu−b−4879' on 'IDP_dMRI_TBSS_MO_Retrolenticular_part_of_internal_
